# Towards an understanding of Internet-based problem shopping behaviour: The concept of online shopping addiction and its proposed predictors

**DOI:** 10.1556/JBA.3.2014.003

**Published:** 2014-02-03

**Authors:** SUSAN ROSE, ARUN DHANDAYUDHAM

**Affiliations:** ^1^Henley Business School, The University of Reading, Greenlands, Henley-on-Thames, Oxfordshire, UK; ^2^Consultant in Addictions Psychiatry, CRI – Northamptonshire, Northampton, UK

**Keywords:** online shopping, compulsive buying, technology addictions, online shopping addiction, problematic online shopping behaviour

## Abstract

*Background:* Compulsive and addictive forms of consumption and buying behaviour have been researched in both business and medical literature. Shopping enabled via the Internet now introduces new features to the shopping experience that translate to positive benefits for the shopper. Evidence now suggests that this new shopping experience may lead to problematic online shopping behaviour. This paper provides a theoretical review of the literature relevant to online shopping addiction (OSA). Based on this selective review, a conceptual model of OSA is presented. *Method:* The selective review of the literature draws on searches within databases relevant to both clinical and consumer behaviour literature including EBSCO, ABI Pro-Quest, Web of Science – Social Citations Index, Medline, PsycINFO and Pubmed. The article reviews current thinking on problematic, and specifically addictive, behaviour in relation to online shopping. *Results:* The review of the literature enables the extension of existing knowledge into the Internet-context. A conceptual model of OSA is developed with theoretical support provided for the inclusion of 7 predictor variables: *low self-esteem, low self-regulation; negative emotional state; enjoyment; female gender; social anonymity* and *cognitive overload.* The construct of OSA is defined and six component criteria of OSA are proposed based on established technological addiction criteria. *Conclusions:* Current Internet-based shopping experiences may trigger problematic behaviours which can be classified on a spectrum which at the extreme end incorporates OSA. The development of a conceptual model provides a basis for the future measurement and testing of proposed predictor variables and the outcome variable OSA.

## INTRODUCTION

Shopping has been defined as: “the process of browsing and/or purchasing of items in exchange for money” (www.businessdirectory.com). It is a process that consists of a number of stages including the search for product information, the processing and assimilation of information in order to evaluate alternative product options, as well as the actual purchase act. A shopping episode may include some or all of these stages and so may or may not include the actual act of purchase. Shopping is today considered both a functional or utilitarian activity as well as a social or leisure activity with hedonistic features (Hirschman & Holbrook, 1982). The enjoyment element has been enhanced by the introduction of large shopping malls offering a range of activities including shopping, eating and entertainment. Authors such as Langrehr (1991) have identified a shift in beneficial experience for the shopper from the purchase item itself to the experience of the shopping process. The highly experiential and sensory nature of shopping provides rewards in itself to the individual, separate from the rewards of the purchase act.

Over the past decade the shopping process has been altered by the advent of the Internet. Internet or online shopping offers a range of benefits in terms of both the information search stage of shopping (Rose & Samouel, 2009) as well as the act of purchase. Cheung, Chan & Limayem (2005) identify a range of internal and external factors that influence consumer purchase behaviour online. These include internal characteristics such as the individual shopper’s attitude to the Internet medium, personal motivations, perceptions of risk and personal innovativeness as well as external benefits that derive from the medium itself such as: convenience, ease of use, perceived usefulness, control and enjoyment (Cheung et al., 2005; Gefen, 2003; Wolfinbarger & Gilly, 2001). The effect of these benefits has been a steady increase in consumer use of online shopping and the consequential value to e-retailers. Given the ease with which the online shopper can now access e-retail websites and purchase online, the penetration of online shopping within the general population is increasing in the UK and other developed countries. Current estimates are that 60% of the UK adult population now take part in online shopping activity (OECD, 2012) and 2012 saw a year-on-year growth of 16% in online sales against an overall increase in retail growth of 4% (Mintel, 2013).

In the UK statistics make dismal reading in terms of negative behaviour leading to addictions. It is estimated that 20% of the population smoke; 200,000 people are in treatment for heroin dependency; over 20% of the adult population is clinically obese and a similar percentage of the population drink alcohol over the recommended limits (Parsons, 2010). Non-chemical addictive behaviours have been referred to as “excessive appetites” and significantly documented in terms of their epidemiology, etiology and comorbidity (Orford, 2006). Research identifies that negative or problem-based behaviours can develop in relation to both consumption (Faber, O’Guinn & Krych, 1987; Hirschman, 1992) and buying (Black, 1996, 2007a, 2007b; O’Guinn & Faber, 1989; Workman & Paper, 2010).

A range of terminology has developed in the domain including: “compulsive buying” (O’Guinn & Faber, 1989; Workman & Paper, 2010), “impulsive purchasing” (Baumeister, 2002); “compulsive consumption” (Faber et al., 1987; Hirschman, 1992); “impulsive spending patterns” (Vohs & Faber, 2003) and shopping addiction (Sussman, Lisha & Griffiths, 2010). The consequences of such behaviour include high levels of debt; negative emotions such as depression or feelings of frustration, shame, guilt and alienation; legal problems; and relationship break-down (Lejoyeux & Weinstein, 2010; O’Guinn & Faber, 1989).

There is evidence emerging that problematic shopping behaviour is now occurring online (Chen, Tarn & Han, 2004). The subject of Internet-based problematic shopping behaviour is therefore an important area of academic research for two reasons. First, given the rapid growth in e-re-tailing, there is currently limited research that identifies the predictive factors of such behaviour (Sun & Wu, 2011). Second, isolation of the predictors of such addictive behaviour would raise awareness amongst the medical profession, e-retailers and consumer groups of this emerging condition. Both of these requirements are met by this paper which presents the theoretical support for a conceptual model of online shopping addiction. The objective of the paper is two-fold. First it reviews the literature in relation to problematic buying behaviour. Second it develops a model that hypothesises seven predictors of the behaviour drawn from prior literature in both the clinical and consumer behaviour literatures.

## METHODS

A selective review of two fields of literature was undertaken: consumer behaviour and clinical addiction. The literature search was conducting using the following key databases: EBSCO, ABI Pro-Quest, Web of Science – Social Citations Index, Medline, PsycINFO and Pubmed. The following key terms were used: “addiction”, “Internet addiction”, “online addiction”, “technology addiction”, “compulsive buying”, “compulsive shopping”, “shopping addiction”, “impulsive buying”, “shopping behaviour”, “online shopping”, “problem online shopping behaviour”, “disordered shopping behaviour”, “ pathological shopping behaviour”. Analysis of the articles was conducted by the authors using the criteria as expressed in the title and objectives of the paper. By this method the authors identified the nature of existing research in the field, epistemological assumptions and methodological approaches. This classification provided a framework through which to analyse the literature.

## SHOPPING BEHAVIOUR

Behaviours are reinforced via the rewards that they elicit. In some respects this aspect of human behaviour is central to survival such as the rewarding nature of food (Davenport, Houston & Griffiths, 2012). Reward sensitivity has been identified as a component of personality and therefore individuals vary in terms of the degree to which they are sensitive to rewards in their environment and the degree to which they are able to control their responses to such rewards. The rewards of shopping have been recognised to extend beyond the actual act of purchase and may include pleasure afforded by the shopping process, the attention and praise of others as well as relief from anxiety or stress (Davenport et al., 2012). Individuals exhibiting problematic buying behaviour have been found to have higher levels of anxiety in response to external and/or internal stimuli and excessive shopping sessions or “binges” have been found to provide quick and ready relief of such anxiety (O’Guinn & Faber, 1989). The terms “compulsion” and “addiction” are both used (often interchangeably) within the literature relating to problematic shopping behaviour, and more recently to online shopping behaviour and so we explore both here.

### Addictive behaviour

Addictive behaviour is a term applied to excessive behaviour that has negative consequences. The word “addiction” is most often used by clinicians to refer to a condition that involves intense preoccupation with the behaviour and leads to physiological changes particularly in the brain. It is characterised by a loss of control and negative outcomes for the individual either psychologically, physically or socially (Sussman et al., 2010). Psychiatrists use the criteria contained in the “Diagnostic and Statistical Manual, Fourth Edition” (American Psychiatric Association, 2000), which is internationally recognised and accepted, to diagnose any disorder with a mental health component and which provides 7 criteria for identification of an addiction dependency.

An addiction is conceptualized as a disorder involving both impulsivity and compulsivity. Impulse control disorders are characterised by two features. Firstly the inability to resist an impulse, drive or temptation even if it is harmful to the individual. Secondly there is a period of tension or arousal prior to the act, relief during the act and regret or guilt after the act (Benson, 2008). People with an impulse control disorder are less likely to have any insight into their behaviour and their ability to resist the behaviour is diminished (Benson, 2000).

### Compulsive behaviour

A compulsion is part of the addictive process. The American Psychiatric Association (1985, p. 234) defines compulsions as “repetitive and seemingly purposeful behaviours that are performed according to certain rules or in a stereotyped fashion”. Such repetitive behaviour is often extreme and takes a ritualistic form. It has the purpose of relieving some form of anxiety or tension within the individual but may result in inappropriate or disruptive consequences (O’Guinn & Faber, 1989; Ullman & Krasner, 1969).

### Problematic buying behaviour

La Rose & Eastin (2002, p. 549) differentiate between “impulsive”, “compulsive” and “addictive” buying as different forms of “unregulated consumer behaviour”. Explanations of compulsive and addictive behaviour have been developed across a range of theoretical approaches including biological (compulsion as an illness or disease), psychological (personality or trait based explanations), or social (social/cultural explanations) (Hirschman, 1992). Compulsive and addictive behaviours have been investigated in relation to consumption which includes both the purchase and use of goods and services (Hirschman, 1992). “Compulsive buying disorder” has been recognised and has been estimated to have a prevalence of 5.8% in the US general adult population (Black, 2007a) and is associated with ways to relieve negative feelings via the reward of short-term gratification (Christenson et al., 1994). As previously stated, the reward element may be derived beyond the actual act of purchase and include aspects of the buying process itself and/or post purchase attention and pleasure (Davenport et al., 2012; Faber, Christenson, de Zwaan & Mitchell, 1995; O’Guinn & Faber, 1989). The comorbidity of compulsive buying with mood disorders such as depression, eating disorders, obsessive-compulsive disorders, substance use disorder and personality disorders have been reported (Lejoyeux & Weinstein, 2010).

O’Guinn and Faber (1989) point out that no single factor explains the etiology of compulsive buying behaviour. Rather they identify a range of factors including levels of arousal (e.g., low boredom or high excitement); release of anxiety or stress; sensation seeking; external environmental stimuli (e.g., the media) or relief from a negative affective state such as low self-esteem. Other identified factors include personality traits (e.g., impulsiveness, instant gratification) and demographics with compulsive shopping strongly linked to women (Workman & Paper, 2010). Black (2007a, 2007b) cites survey studies in which the prevalence rate of compulsive buying disorder amongst women is as high as 80% to 94% although there are suggestions that such findings may be an artefact of sampling methods. However, gender differences have been identified with men more likely to be addicted to drugs, gambling and sex (Holden, 2001), whilst women are more likely demonstrate disorders in relation to eating and shopping (referred to as “mall disorders”) (Davenport et al., 2012). Legendary female excessive shoppers have included two US First Ladies: Mary Todd Lincoln and Jacqueline Kennedy Onassis, Imelda Marcos (Former First Lady of the Philippines) and Princess Diana in the UK (Black, 1996, 2007a).

O’Guinn and Faber (1989) note that, our view of the severity and consequences of compulsive shopping is dictated by how the behaviour is perceived by society. They suggest that at one extreme level compulsive buying may be viewed by society as a “crime” whilst at another merely a “bad habit”. An obsessive shopper may be able to accommodate their behaviour within their everyday life and whilst it may appear worrying to others, it may not necessarily create negative consequences for the individual.

Lejoyeux & Weinstein (2010) suggest that similarities exist between compulsive buying and addiction in terms of the clinical characteristics. However, Sussman et al. (2010) provide a clear distinction between compulsive and addictive behaviour by identifying the characteristic differences. An addiction will typically involve a lot of time spent on the part of the individual thinking about engaging in the behaviour and is therefore typified by intense preoccupation that is beyond the need to release immediate anxiety as typically found in compulsive disorders. Rather addiction is characterised by loss of control or an inability to freely decide whether to engage in the behaviour or not. The individual is unable to predict when the behaviour may occur, how long it will last or when it will stop. Finally an addiction will have negative long-term effects for the individual that may include detrimental effects upon finances, social relationships, the ability to work effectively or to lead their lives appropriately. Edwards (1993) proposes that such shopping behaviours range across a continuum from normal behaviour where purchase is according to the individual’s needs and wants through to addictive with a severe lack of control. Given the recognised negative consequences of online shopping addiction for consumers, we focus upon this far end of the continuum in our work. Taking the Sussman et al. (2010) definition of addiction, we now move to discuss its application within online shopping.

## DEVELOPMENT OF A CONCEPTUAL MODEL OF ONLINE SHOPPING ADDICTION

It has been recognised that a society is vulnerable to addiction at the stage when a new substance or behavioural activity is first introduced into the culture. For example, the introduction of alcohol into native cultures that have no prior exposure to the substance demonstrate higher prevalence of addiction to the substance compared to those in established societies (Orford, 2006). Our exposure time to the Internet is approximately 15 years and therefore adaptation to online behaviour is still in its infancy and it can be argued consumers are vulnerable to its influence. New technologies have the ability to influence subjective experiences so powerfully as to make them potentially addictive activities (Orford, 2006). The Internet is one such technology and there are several subtypes of Internet related problem behaviours that have emerged such as online sexual addiction, social media addiction, online gaming and gambling addictions that combine both pre-established addictions with Internet addiction (Griffiths, 1995). It has been questioned whether Internet addictions actually do exist or whether the Internet is the medium through which pre-existing addictive behaviour is carried out. This view is proposed by authors such as Griffiths (2000) who suggests that technological addictions should be viewed as a subset of behavioural addictions which of themselves demonstrate the core elements of addiction. Therefore true Internet addictions may not be highly prevalent (Widyanto & Griffiths, 2007).

The literature in relation to problematic online shopping behaviour is currently limited and most often discussed within the context of broader Internet dependency or addictions (Chen et al., 2004). Sun and Wu (2011) link problematic online buying behaviour to addiction to the Internet itself. They conclude that emotional instability and materialism have positive effects upon Internet addiction, which in turn positively influences impulsive online buying. Materialism and impulsiveness have been linked to technology addiction for example in young people and cell-phone use (Roberts & Pirog, 2012). La Rose and Eastin (2002) found evidence of unregulated online buying amongst college students and evidence for the role of poor self-regulation in influencing this behaviour. They propose that this irrational, lack of control, element of online shopping can be a stronger determinant of online shopping behaviour than rational, economic considerations.

Other studies have focused upon the consequences of OSA such as that by Lo and Harvey (2012) who look at the ways in which compulsive shoppers differ to normal shoppers in terms of their buying process and their emotional responses to the consequences of their buying. The study utilised an experimental design which involved the observation of online shoppers as they performed various shopping tasks such as searching, adding items to the shopping cart and payment. Data was captured both during the shopping process and following the experience which showed distinct differences in compulsive and non-compulsive shoppers. For example compulsive shoppers did not check product information in such detail and were less concerned about over-spending identified by credit card usage. The authors concluded that the compulsive shopper is addicted to the process of shopping itself, experiences cravings to shop but tends to ignore the consequences of satisfying such cravings.

**Figure 1.**
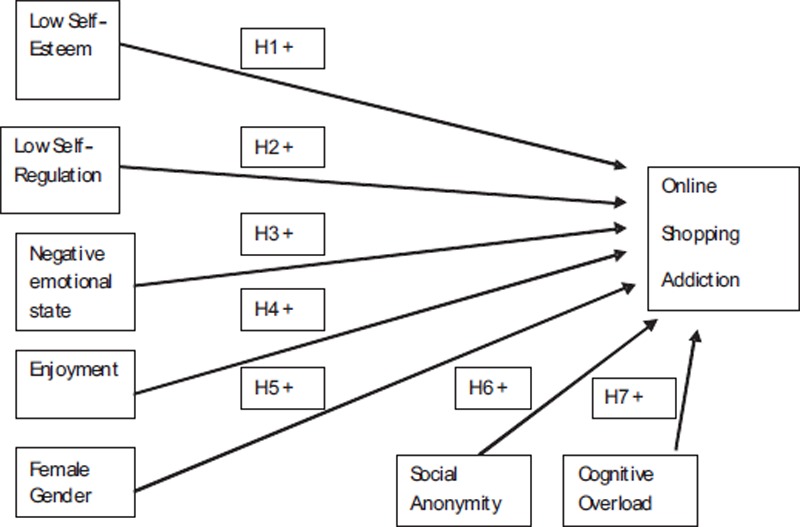
A conceptual model of online shopping addiction (OSA)

Given the limited understanding of the predictors of OSA, we propose that insights will be found in both existing theories of addictive/compulsive buying behaviour and by identifying specific features of the online retail medium which may encourage OSA. The model therefore incorporates recognised predictors of addictive behaviours in general: *low self-esteem, low self-regulation, negative emotional state* and *female gender* as well as predictors specific to the online retail medium: *enjoyment, social anonymity* and *cognitive overload.* Figure 1 below presents our conceptual model of OSA and we discuss the theoretical support for the hypothesised relationships between OSA and the 7 proposed predictors.

### Low self-esteem

The effect of low self-esteem is consistently reported in terms of compulsive and addictive behaviour (Davenport et al., 2012; Hirschman, 1992; O’Guinn & Faber, 1989). Compulsive buying is an attempt to relieve feelings of low self-esteem (Jacobs, 1986). Low self-esteem is relieved by the reward or outcome of the repetitive behaviour. Davenport et al. (2012) point out that it may not be the goods purchased but involvement in the buying process that relieves anxiety and feelings of low self-esteem. We therefore hypothesise that individuals with low self-esteem will find the process of online shopping provides relief from such feelings and therefore this factor will have a direct effect upon OSA.

### Low self-regulation

Difficulties with control are strongly linked to compulsive and addictive behaviour (Baumeister, 2002; Hirschman, 1992; Workman & Paper, 2010). Baumeister (2002) defines self-control as the individual’s capacity to alter their own state or responses to stimuli and uses the term interchangeably with self-regulation. Self-regulation has been recognised within the consumer behaviour literature. Vohs and Faber (2003) view self-regulation as being managed by a limited set of resources that the individual draws on to control their responses. These resources include cognitions, emotions or behaviours. They propose that each act of self-control depletes the resources and therefore reduces the individual’s overall capacity for self-regulation. Sun and Wu (2011) recognise the relationship between Internet addiction and lack of self-control and suggest that a continuous exposure to the online environment that encourages lack of self-control depletes the individual’s resource capacity for self-regulation. La Rose and Eastin (2002) identify that whilst there are features of online retail websites that encourage self-regulation (e.g., shopping carts to contain products before purchase and therefore time to consider; search engine opportunities to search and evaluate information; or past purchase history to trigger awareness of buying behaviour) these were found to be outweighed by retail website features such as advertising pop-ups, timed discount offers, vivid interactive graphical displays of products, or ‘one click’ purchases, all of which encourage purchase and weaken self-regulation. In their study La Rose and Eastin (2002) found a direct relationship between deficient self-regulation and online shopping activity and we similarly include this effect within our conceptual model.

### Negative emotional state

Emotion has been identified as a factor in the continued use of technology (Oritz de Guinea & Markus, 2009). Baumeister (2002) suggests that at times of emotional distress an individual is more likely to loosen self-control and act in an impulsive way in order relieve such feelings. Shopping has been recognised to ease anxiety and stress and therefore a shopper in a negative emotional state is more likely to act impulsively and excessively (Davenport et al., 2012). We hypothesise that the ease of access and instant gratification of the e-retail medium provides an ideal environment for reduction of negative emotional state. Therefore negative states are a driver of OSA.

### Enjoyment

Psychologically enjoyment has been identified as a hedonistic emotion which positively motivates physical activity and has been linked to website experience (Lin, Gregor and Ew-ing, 2008). Davenport et al. (2012) identify “reward sensitivity” as an influence upon compulsive buying proposing that an individual who is highly sensitive to rewards will respond to enjoyable stimuli such as food or shopping. Hedonic motivations have been identified in relation to shopping (Arnold & Reynolds, 2003). Enjoyment has been found to be a determinant of an individual’s likelihood to continue to shop (Hart, Farrell, Stachow, Reed & Cadogan, 2007) and pleasure is identified as a rewarding aspect of compulsive buying behaviour (Davenport et al., 2012). Similarly enjoyment has been identified as a motivator of online shopping (Wolfinbarger & Gilly, 2001). Lejoyeux and Weinstein (2010, p. 249) report that positive feelings of pleasure or excitement (often referred to as “a high” or “a rush”) are associated with compulsive buying. We therefore incorporate this into our model and hypothesise that enjoyment is a key factor in the development of OSA.

### Gender

Pratarelli and Browne (2002) provide evidence that certain addictions may vary by gender. There is consistent evidence that women are more likely to demonstrate compulsive and addictive buying behaviour (Black 2007a; Christenson et al., 1994; Davenport et al., 2012; McElroy, Phillips & Keck, 1994). This may be explained by the fact that in Western countries females are socialized and expected to do most of the household shopping. From an early age young females learn that shopping and buying are activities that can be used to feel better and therefore can be used to combat negative mood states (Benson, 2008). Whilst excessive Internet usage has been found to be male dominated particularly in the area of gaming and gambling (Mottram & Fleming, 2009), we hypothesise that the female gender variable is likely to be a stronger predictor of OSA than being male, in line with prior clinical findings.

### Social anonymity

As stated, shopping is traditionally a social activity involving personal interaction with others (other shoppers and/or retail staff). Online shopping is (most often) solitary and a key feature is the social anonymity of the shopping environment. Lejoyeux and Weinstein (2010) suggest that compulsive buying may be prompted by the online retail environment because of the social anonymity that allows the shopper to keep their buying behaviour private from others, such as their family. Added to the effect of anonymity, Sun and Wu (2011) suggest that an appeal of online shopping is that the individual may feel less inhibited about their shopping when they are not visible to others. Disinhibition has been identified as a distinctive feature of Internet behaviour encouraging many forms of inappropriate behaviour such as intimate self-disclosure on social media sites; “flaming” or negative comment about others; through to activities such as bullying or the use of pornography sites (Joinson, 2007). Such behaviours come about due to two factors. First, the lack of concern by the individual about self-presentation (how they appear to others) and second how others may judge them due to reduced social cues in the virtual environment (Joinson, 2007). We hypothesise that the social anonymity of the online environment and subsequent disinhibi-tion encourages inappropriate excessive shopping behaviour due to the absence of the regulation of normal shopping environmental cues such as the response behaviours of other shoppers or retail staff.

### Cognitive overload

Compulsive buying behaviour has been linked to arousal and the effect of the external environment (O’Guinn & Faber, 1989). Online retail sites satisfy the need for arousal among shoppers by the dynamic nature of the medium. This includes devices such as graphic displays, interactive dialogue and “pop ups” providing product information or notification of special offers. Such frequent and constantly changing stimuli provide repeated stimulation and temptation potentially creating cognitive overload for the individual. Increases in the cognitive load of the individual in one area can overwhelm self-control in another leading to lack of willpower. As discussed earlier, self-control is managed by a limited set of resources and authors such as Muraven & Baumeister (2000) have applied a ‘limited resources model’ to explain the concept of self-control. They propose that the resource needed for an individual to exert self-control is limited and the resource is partially depleted by the act of self-control itself. Increased cognitive load, which similarly depletes resources, has been found to make temptation harder to resist (Fudenberg & Levine, 2006). We therefore hypothesise that the cognitive stimulation of online retail websites will increase cognitive load leading to lack of self-control and so have an effect upon OSA.

### The dependent variable: Online shopping addiction

Our start point to identify the components of OSA and therefore definition criteria is to look at the literature regarding technological addiction. The Internet as a form of technology addiction is now well researched (Chen et al., 2004; Leung, 2004) particularly in the areas of online gambling and gaming (Griffiths, 2009; Wood & Griffiths, 2007). Griffiths (1995, p. 15) first introduced the term “technology addictions” which he defines as “non-chemical (behavioural) addictions which involve human-machine interaction”. As stated, such addictions may combine with other categories of addictive behaviour such as gambling, sex or shopping. In developing a definition of technological addiction, Griffiths (1995) cites Marlatt, Baer, Donovan and Kivlahan (1988) in terms of defining addictive behaviour as characterised by loss of control, an inability to withdraw from the behaviour despite attempts, and long-term negative consequences. Griffith (1995) draws on the clinical criteria for established addictions (DSM4) to develop an understanding of technological addiction and development of criteria. The application to Internet behaviour is supported by subsequent studies (Meerkerk, Van den Eijnden, Vermulst & Garrelsen, 2009) and we similarly propose that the Griffiths technology addiction criteria be adapted to online shopping to measure the components of OSA.

## CONCLUSIONS

This article reviews literature on compulsive and addictive shopping and the emergent literature in relation to problematic online shopping behaviour. The contribution of this review is that it fills a gap in the literature in terms of the identification of potential predictors of online shopping addiction. Seven predictor variables are proposed to influence the likely development of OSA which includes: *low self-esteem, low self-regulation; negative emotion, enjoyment, gender, social anonymity* and *cognitive overload*. The dependent variable of OSA is predicted to have six component features that include: *salience, euphoria, tolerance, withdrawal symptoms, conflict* and *relapse.* Development of the model helps both clinicians and retailers to recognise the pre-conditions for the development of addictive consumer behaviour when shopping online. Whilst not all of the proposed predictors of OSA are within the control of e-retailers, the research seeks to shed light on an important aspect of consumer retail behaviour. Further research is called for in order to development measurement scales and testing of the proposed conceptual model.
